# Structural and Functional Responses of Small Mammal Communities to Land Abandonment in a Region of High Biodiversity

**DOI:** 10.3390/ani15131857

**Published:** 2025-06-24

**Authors:** Anamaria Lazăr, Marcela Alexandra Sandu, Ana Maria Benedek, Ioan Sîrbu

**Affiliations:** 1 Faculty of Food and Tourism, Transylvania University of Braşov, Eroilor Bd. 29, 500362 Braşov, Romania; 2 Doctoral School in Ecology, Faculty of Biology, University of Bucharest, 91-95 Splaiul Independenței, 050096 Bucharest, Romania; marcela-alexandra.sandu@s.unibuc.ro; 3 Biology and Ecology Research Center, Faculty of Sciences, Lucian Blaga University of Sibiu, 5-7 Rațiu Street, 550012 Sibiu, Romania; ioan.n.sirbu@ulbsibiu.ro

**Keywords:** agricultural land-use change, arable land, pastures, fallowing, functional traits, rodents

## Abstract

Small mammals are common in farmland, where their communities are affected by agricultural management. However, so far, no clear patterns have emerged, its effect varying in accordance with the ecological context, spatial scale, and geographic area. We aimed to assess whether the discontinuation of land cultivation and pasture grazing leads to significant changes in the abundance, diversity, and composition of small mammal communities. These were surveyed in transects of live traps set in used and abandoned arable fields and pastures in highly patched agricultural landscapes in Transylvania (Romania). Farmland abandonment was positively related to species richness, taxonomic and functional diversity, and abundance. Its effect was stronger in pastures, where intensive grazing is a limiting factor for small mammals. Functional trait composition was also sensitive to fallowing and abandonment of grazing, which promote diurnal activity, broader niches, and lower fertility. In conclusion, small mammals benefit from the maintenance of uncultivated plots and low numbers of grazing livestock, which we recommend as management strategy in traditional mosaic landscapes, to support taxonomic and functional biodiversity with implications in ecosystem service functionality. Our results also revealed more diverse communities than those showcased by similar studies in central and western Europe, with similar overall abundances.

## Simple Summary

Our study conducted in southeastern Transylvania (central Romania) is aimed at assessing the effect of farming abandonment on the diversity, abundance, and community composition of small mammals, a functional group of animals having important ecological functions in terrestrial ecosystems. The research area features a mosaic agricultural landscape characterised by a variety of small-sized arable fields and extensive pastures. This mosaic allows the coexistence of numerous species, thus enriching the regional diversity of small mammal communities, which is higher than in agricultural landscapes in western and central Europe. The abandonment of agricultural land, a result of the depopulation of villages and the economic inefficiency of small-scale farming, was associated with an increase in small mammal abundance and diversity, as well as a change in the functional trait composition. Our results show that local temporary abandonment of arable land and pastures may help preserve and boost small mammal diversity and suggest that agricultural management strategies that conserve the mosaic of habitats are essential for maintaining biodiversity in agricultural landscapes.

## 1. Introduction

Farmland is the major land use in Europe, and consequently, European biodiversity is contingent on the management of agricultural habitats [1,2,3]. To achieve common agricultural policy (CAP) objectives, such as increasing agricultural productivity and individual earnings of agricultural workers, major changes were imposed, and have been implemented in farmland management [2,4]. These changes have resulted in larger parcel sizes and the loss of seminatural elements, such as hedges and field margins. The farm management upheaval coincided with the loss of ecological heterogeneity [5,6] in agricultural landscapes, and the subsequent decline of many specialist farmland species, both invertebrate and vertebrate, including small mammals [7,8]. However, there are regions where agricultural disturbance has been reversed as agrarian activities have ceased. Arable land undergoes fallowing when being set aside or abandoned. Set-asides are plots temporarily not cultivated, which were first regulated in Europe by the European Economic Community in 1988 as an incentive scheme. This was mainly aimed at reducing produce surpluses and soil erosion, while also promoting wildlife biodiversity [9,10]. In 1992, a 15% set-aside (10% as of 1996) of farmland became mandatory [11]. Especially birds, but also insects and small mammals benefit from the set-asides in intensive agriculture landscapes, while in complex landscapes, their beneficial effects were found to be negligible [10,11]. Despite its numerous environmental benefits, the EU set-aside scheme was abolished entirely in 2008, on economic grounds. However, this scheme is not the sole reason for the increase in fallowing. In some areas, such as those surveyed in our study, land fallowing results from the depopulation of villages and the economic inefficiency of traditional small-plot farming, which still dominates the landscape. Moreover, in pastures, ceasing or reducing livestock grazing also induces changes in the environmental conditions, reversing them towards a more natural state.

In 2007, Romania joined the European Union and the CAP regulations. Thus, the agricultural landscape has been altogether reshaped by both the intensification of agriculture and the abandonment of agricultural land. While this has had severe consequences for many species dependent on farmland habitats [12,13,14], studies focusing specifically on the effect on small mammals are altogether absent. In Romania, individual farms span, on average, over 2.15 hectares, divided into 3.7 plots, while in Transylvania—the region in Central Romania—the surface of farming plots is below the national average [15]. Here, traditional farming landscapes are still widespread, continuing to support high biodiversity [15,16,17,18,19].

During most of the growing season, farmland provides good food resources for many species, including small mammals [20], with rodents and shrews being a common presence in agricultural habitats [3,8,17,20]. Here, rodents are generally labelled as pests, as they cause crop damage. For this reason, they are generally not addressed in biodiversity studies, despite constituting important elements in food webs, as the main prey biomass [21] for vertebrate predators, directly influencing their abundance and diversity [22]. Consequently, small mammals affect a wide range of species at higher trophic levels, making them paramount to conserving many mammalian and avian predators [21].

Farming practices and land use strategies cause stress to small mammals by removing shelter, food, breeding, and overwintering sites [2]. By contrast, farmland abandonment has generally been equated to high habitat heterogeneity, which boosts small mammal abundance and diversity [23]. Unfortunately, few studies have addressed this aspect specifically [9,11], most data coming from studies on various types of land use [6,23].

More recently, studies have shown that the effect of habitat structure is not only noticeable at the community composition and abundance level, but arguably also influences the functional traits of rodents [24,25]. Intense human activity has driven various animal groups to adapt to habitat changes by changing migration patterns, phenotypic plasticity, and body size [24,26]. The high variability of functional traits, such as body size, trophic level, reproduction rate, and activity, has been directly linked to highly heterogeneous landscapes, including aggregated forest patches and woodland with dense shrub layers [25]. However, studies that consider the functional traits of small mammals in agricultural landscapes are scarce, this approach being in its infancy.

In this study, we evaluated the abundance, species richness, taxonomic, and functional diversity, as well as the community species and functional composition as parameters of small mammal assemblages. It was conducted in two agricultural landscape mosaics of arable land and pastures. We aimed to test the following hypotheses: (1) since abandoned land has more heterogeneous vegetation and is subjected to lower human disturbance, we predicted higher diversity and abundance in abandoned compared to used farmland; (2) because of the great differences in habitat features and the history of disturbance, the changes in community composition following abandonment are different between arable land and pastures; (3) because of shrub encroachment on abandoned pastures, the grassland specialist *Microtus arvalis* (common vole) is replaced by the forest specialist *Apodemus flavicollis* (yellow-necked mouse). We also aimed to identify which species are associated with used and abandoned arable land.

## 2. Materials and Methods

### 2.1. Study Areas

**Figure 1:**
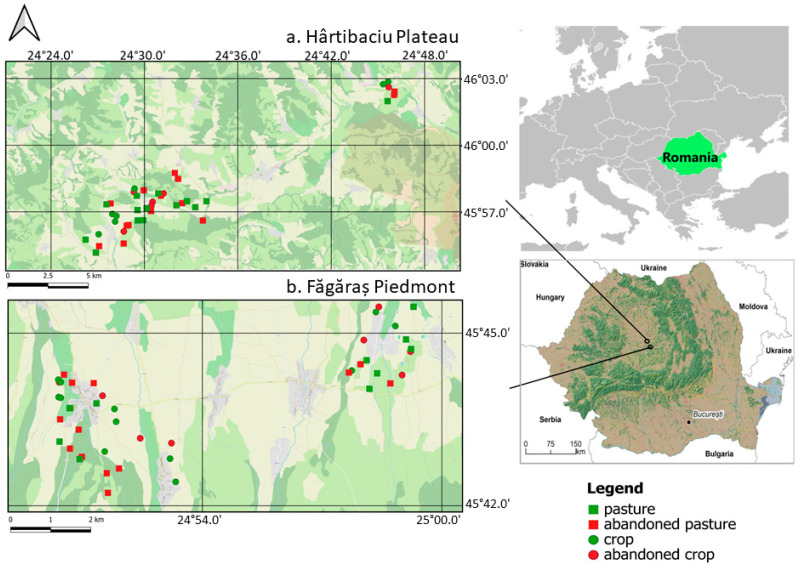
Map of the study areas: (**a**) Hârtibaciu Plateau and (**b**) Făgăraș Piedmont and the location of the trap transects. Maps were drawn in QGIS, version 3.36.1 [28].

We conducted small mammal trapping in two Natura 2000 sites in southern Transylvania (Romania), designated under the European Union Directive on the Conservation of Wild Birds: ROSPA0099 Hârtibaciu Plateau, between 45.9365° and 46.04598° N and 24.45767° and 24.76733° E, between 410 and 580 m a.s.l., and ROSPA0098 Făgăraș Piedmont, in the Făgăraș Depression, between 45.740128° and 45.685142° N and 24.83145° and 25.006792° E, between 565 and 600 m a.s.l. (Figure 1a,b). The present study is based on the field data collected within the framework of the project LIFE08 NAT/RO/000501, “Conservation of *Aquila pomarina* in Romania”, during the small mammal survey which aimed to evaluate the prey availability for this raptor bird in the two study areas.

In the Hârtibaciu Plateau (Figure 1a), the multi-year average monthly temperatures recorded in the town of Sighișoara, situated slightly beyond the northern extremity of the plateau, range between a minimum of −8 °C in January and a maximum of 18 °C in July. In the study area, the average annual precipitation increases with altitude from 600 mm in the extreme northwest to 700 mm in the extreme east. The rainiest season is summer (June–August), with 250–300 mm of rainfall [27]. The land use mapping performed within the LIFE project revealed that, in the Hârtibaciu Plateau, the crops—mainly cereal (maize and wheat)—covered between 2% and 34% of the open land surface in different parts of our study site. Based on our observations in the field, maize is sown in widely spaced rows, often invaded by weeds. In some plots, weeds are either removed mechanically or by hand, or herbicides are used, while other plots are left weedy. Land fallowing induces significant changes in the vegetation, favouring the establishment of invasive species, such as *Solidago canadensis*, *S. gigantea*, and *Erigeron annuus*, widespread mostly in moist habitats along rivers and ditches. Fallow land commonly features tall vegetation throughout the summer and autumn. Pastures cover the largest surface among the open habitats and are often overgrazed mainly by sheep. Where the ligneous plants are not removed (grazing alone does not prevent the encroachment of shrubs; they need to be removed mechanically), pastures are quickly invaded by shrubs and trees, inducing a secondary succession to broadleaf forests.

**Figure A1:**
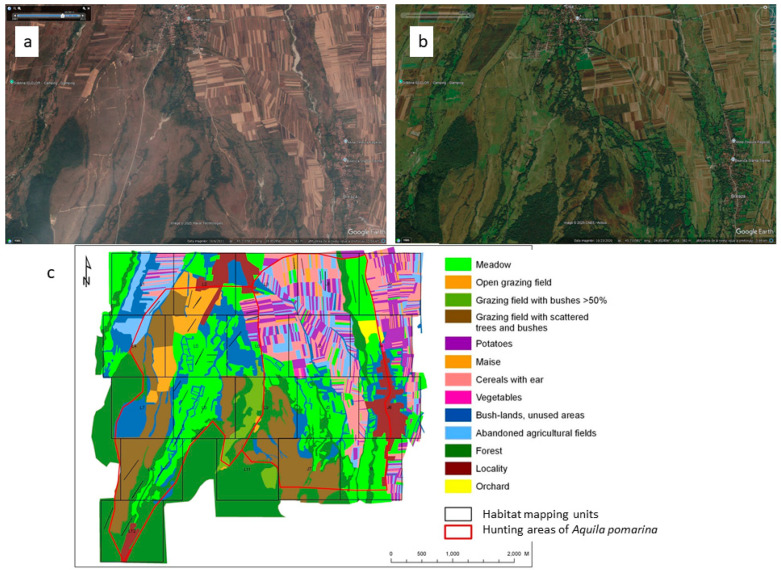
Aerial view of the study area in Făgăraș Piedmont: (**a**) during the study period, in October 2011 and (**b**) ten years later, in September 2021, illustrating the maintenance of the high level of fragmentation of the arable land. (**c**) Represents the land uses in the research area during the study period. Aerial photographs are from Google Earth [66].

The researched area in Făgăraș Piedmont (Figure 1b) has a flat relief with an average slope of 2.5°. It has a well-defined geographical individuality, clearly distinguished from the neighbouring regions. The multi-year average monthly temperature values recorded in the town of Făgăraș, situated at the eastern limit of the piedmont, spans between a minimum of −4.6 °C in January and a maximum of 18.7 °C in July. The relative air humidity is high, with an annual average of 72%; the minimum values are recorded in July (68%) and the maximum in December (75%). The yearly average rainfall is generally high (about 800 mm), with the highest amounts of water falling between May and August (59.7%) and the maximum rainfall in July (about 1300 mm) [29]. Based on our own observations in the field, agriculture was practised on small plots (<1.5 ha), giving the landscape a mosaic-like appearance. Although 15 years have passed since our field survey, the patchy nature of the landscape has not changed, as satellite images of the area show (Appendix A, Figure A1a,b). Crops, especially potatoes and maize, are interspersed with patches of grassland, pastures, and abandoned arable fields in the early stages of vegetation succession, where *Chenopodium album*, *Polygonum aviculare*, *Artemisia annua*, *Amaranthus retroflexus*, and *Ballota nigra* predominate. The area is crossed from south to north by watercourses flanked by woody vegetation, providing connectivity with neighbouring forest habitats. The agricultural practices entail annual crop rotation. Grazing is limited to cattle in a local system, with animals moving daily between the pasture and the local households. Abandoned pastures occur due to the gradually decreasing number of cattle the villagers own. Thus, pastures far from the village are no longer frequented by livestock.

**Figure 2:**
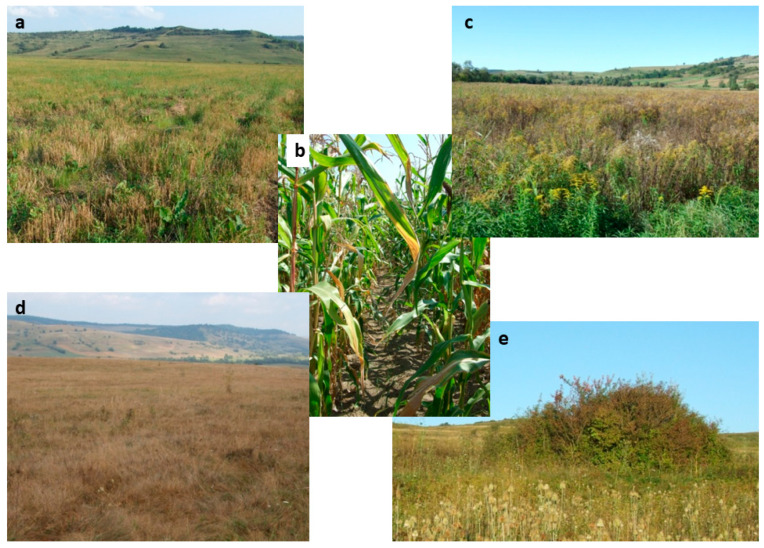
The typical habitats researched during our survey: (**a**) wheat field after harvest in late summer; (**b**) maize crop in summer; (**c**) fallow field in early autumn; (**d**) grazed pasture in autumn; (**e**) shrub encroachment in abandoned pasture.

In this study, we consider the data collected from farmland habitats classified based on two characteristics at the moment of survey—land use (arable land and pasture) and abandonment (in use and abandoned). We defined abandonment by the lack of agricultural activities during the year of survey, regardless of the time of abandonment or its temporary or permanent character. The four resulting habitat types include cultivated fields (crops) (Figure 2a,b), abandoned arable land (fallow) (Figure 2c), grazed pastures (Figure 2d), and abandoned pastures (Figure 2e). Cultivated fields included wheat (Figure 2a), corn (Figure 2b), and potato crops (the last only in Făgăraș Piedmont). Although well represented in the surveyed areas, we did not include in our analysis alfalfa fields because of their perennial nature, which implies the absence of annual soil disturbance by tillage, making them not comparable with annual crops. Although we do not know the time of fallowing onset, we can say that it was recent (less than five years), as they were all in an early stage of succession. Based on our field observations, most fallow plots were covered by tall and dense herbaceous vegetation (Figure 2c), but none presented shrub encroachment. In our research areas, the cessation of land cultivation is temporary, depending on the social and economic situation of the villagers. Pastures were extensive and had low vegetation, sometimes with isolated trees or shrubs, often with signs of overgrazing (Figure 2d). Abandoned pastures were not fenced; therefore occasional grazing occurred, but shrubs were not removed; as a result, shrubs were always present, their growth depending on the time of abandonment. Based on the cover and height of the shrubs, we estimate that, in most pastures, abandonment occurred less than 10 years earlier. The herbaceous layer was also higher compared to the grazed pastures, comprising unpalatable tall species, such as *Dipsacus* spp., *Cirsium* spp., and *Carduus* spp. (Figure 2e).

### 2.2. Small Mammal Trapping

Field campaigns were conducted for two consecutive years, between August and October 2010 and June and October 2011. Because we were interested in the response of small mammals to abandonment at the community level, we sampled individual plots using transects (trap lines) instead of trapping grids, as the former are more suitable for long and narrow cultivated plots. We live-trapped small mammals using artisanal single-catch plastic box traps (18 × 8 × 6 cm) set in lines of 30 traps placed at 10 m intervals, one trap per point. Each transect, measuring 300 m, covered only one individual field of the same land use type and did not reach the habitat edges. The location of transects was chosen by stratified random sampling, the number of transects in each habitat being proportional to its representation within the study areas. The distance between transects varied, usually between 100 and 500 m. The traps were baited with sunflower seeds and apple slices. No pre-baiting was performed. In 2011, the plots were sampled twice, once in the summer (June–July) and once in the autumn (end of August–September). Habitat types were assigned to the sampling period randomly, with each habitat type sampled at least once each month. Five transects were set simultaneously, and the traps were checked for three consecutive days at dawn. Trapping amounted to 63 days of field work (15 in the autumn of 2010, 24 in the summer of 2011, and 24 in the autumn of 2011). We set in all a total of 94 transects, 47 in Făgăraș Piedmont (9 in 2010 and 38 in 2011) and 47 in Hârtibaciu Plateau (15 in 2010 and 32 in 2011) (Table S1), resulting in a total trapping effort of 6707 effective trap nights (Table 1), calculated as the number of transects (94) multiplied by the number of traps per transect (30) and the sampling nights (3). We excluded the non-functional traps (1753), which were disturbed or destroyed by animals or people, or accidentally closed by animals, rain, or wind.

The captured specimens were taxonomically identified to species level based on morphological and biometrical traits, according to [30]. They were marked by fur clipping on the rear part of the back and released at their trapping site. Recaptures were not considered in the data analysis.

The trapping and handling of the animals within the Natura 2000 sites were carried out at the invitation of the Environmental Protection Agency of Sibiu, using the protocols on the trapping and handling of animals developed and approved by this institution. All protocols are in line with EU Council Directive 86/609/EEC on the experimental use of animals. Subsequently, the protocol was also approved by the Biomedical Research Ethics Committee of the Lucian Blaga University of Sibiu (approval 9/26 May 2021).

### 2.3. Functional Traits

Determining values of morphological or life history traits, diet, or activity patterns for each species is the aim of standalone studies. These trait values are subsequently synthesised in databases containing numerous species, which are made available for use by the researchers. Despite the obvious shortcomings of using trait values from databases (which give one value per trait for a species, thus not accounting for their variability, which may be significant and case-dependent), most often this is the only way to answer research questions involving the functions of animal communities. To evaluate the functional responses of small mammal communities to farming management, we used traits that were evaluated based on original data from Romania (those concerning adult size and habitat niche) or were extracted from the COMBINE database [31]. For mammals, this is the most comprehensive and widely used trait database. We selected 12 traits concerning the following: 1. Body size: weight—adult body weight (g), body length—adult body length (mm), weight birth—neonate weight (g); 2. Life history: fertility—mean number of young born per year by the female, first reproduction—age of first reproduction (days), gestation—gestation length (days), generation—generation length (days); 3. Diet: diet niche—Levins niche width index, trophic level—level of carnivory expressed as an ordinal variable (1—herbivore, 2—omnivore, 3—carnivore); 4. Habitat and activity: habitat_niche—Levins niche width index, T/B_length—tail to body length ratio (a proxy for arboreality versus fossoriality), and activity—degree of diurnality expressed as ordinal variable (1—mainly nocturnal, 2—crepuscular or cathemeral, 3—mainly diurnal). Functional traits were used to calculate the community weighted mean (CWM) and functional diversity. The CWM expresses the functional structure of communities. Each community is characterised by the average of trait values, weighted by the species’ abundance [32]. The use of the CWM represents the community-based approach of the trait–environment studies, and it allows for the assessment of how the community trait composition is affected by the environment [32]. Functional diversity represents the variability in traits within the community, which influences how the ecosystem functions. It can be evaluated either for the entire community, considering all the traits together, or for each trait separately (resulting in one diversity value for each trait in each community). In the present study, both approaches were adopted.

### 2.4. Data Analysis

We assessed the effect of the abandonment of arable land and pastures on small mammals by comparing annual crop habitats with fallow habitats and grazed pastures with abandoned pastures. We used the following response variables for small mammals: total abundance, observed and estimated species richness, taxonomic and functional diversity, and taxonomic and functional community composition. As a proxy for abundance, we used the number of captured individuals but, since the trapping effort strongly influences capture success, we included it in the models or used it to calculate the trapping rate—the number of captured individuals per 100 effective trap nights. As a measure of species richness, we used the observed (S) and estimated (Chao) number of species captured per transect; for taxonomic diversity, we used the Shannon index (H); while for functional diversity, we used Rao’s quadratic entropy (Rao), which describes the trait diversity among species coexisting within a given community [33]. Chao and H were calculated using the vegan package [34] in R version 3.6.1 [35], and Rao was calculated using Canoco 5.12 [36], both for overall and for each trait separately. To compare the overall diversity in the abandoned and used land, and to evaluate the species saturation of samples, we constructed the rarefaction curves for each category using the function iNEXT in the iNEXT package [37]. Because of the variation in sample size (different trapping efforts among transects), we used the individual-based approach to construct rarefaction curves. To test the differential effect of grazing abandonment on the two specialist species, *M. arvalis* and *A. flavicollis*, we used Fisher’s exact test for independence of categorical variables.

We used generalised linear mixed models (GLMMs) with a negative binomial distribution (because of significant overdispersion) to test the effect of abandonment on small mammal abundance. We included the number of captured individuals as the response variable and the trapping effort as an offset. We considered spatial dependence between the transects, including sampling stations, as a random factor and temporal dependence between the transects, including the month, as a random factor. We also tested the effects of year and survey area, but these were not significant, so we did not include them in the final models. To test the differential effect on the two farmland uses (crop and pasture), we also included the interaction between abandonment and use. When testing the effect of abandonment on diversity measures, random effects were not significant; therefore, we constructed linear models, their assumptions being met. We report only the best model, found by comparison using the likelihood-ratio test. In the GLMMs, the explained variation for the best model was expressed by the conditional and marginal pseudo-R^2^ statistics based on Nakagawa et al. [38] computed in the MuMIn package [39]. The marginal R^2^ represents the variance explained only by the fixed part of the model, while the conditional R^2^ is interpreted as the variance explained by the entire model, including both fixed and random factors. For the linear models, we reported the adjusted coefficient of determination (R^2^ adj).

The effect of land abandonment at the community level was analysed using Canoco 5.12 software [36], performing three partial redundancy analyses (RDA) with the species composition, the CWM, and the functional diversities as response variables, land use type and abandonment as predictors, and station and month as covariates. Rare species (singletons and doubletons) were not included in the analyses of species composition but were considered for calculating the CWM and functional diversities. Response data were log-transformed by y’ = log(y + 1). The significance of ordination axes was tested by the Monte Carlo permutation test with 999 permutations per test. Permutations were restricted to blocks defined by the survey month and station. The significance of the response of individual species to the selected predictors was evaluated through t-value biplots, which approximate the t-values of the regression coefficients of multiple regression with the particular species as the response variable and all the habitat types as predictors, revealing statistically significant pairwise relationships between each response variable and each predictor [32].

Because the trapping methodology (type of traps and bait) was not best suited for insectivorous shrews, to avoid biased results, we performed all the analyses also only on the rodent data.

## 3. Results

### 3.1. Small Mammal Species Composition, Richness and Diversity

During our study, we trapped 991 individuals from 16 small mammal species (Table 2). *Microtus arvalis* (common vole, 41.2%, SE = 1.6) and *Apodemus agrarius* (striped field mouse, 35.5%, SE = 1.5) were the most abundant. Seven species were either singletons (*Crocidura leucodon*—bicoloured white-toothed shrew, *C. suaveolens*—lesser white-toothed shrew, *Muscardinus avellanarius*—hazel dormouse, *Clethrionomys glareolus*—bank vole, *Micromys minutus*—harvest mouse, and *Rattus norvegicus*—Norway or brown rat) or doubletons (*Sorex minutus*—pygmy shrew), and only two of them (*C. leucodon* and *S. minutus*) were found in used farmland. *Arvicola terrestris* (water vole) was captured in low numbers but was found both in used and abandoned farmland (Table 2).

**Figure 3:**
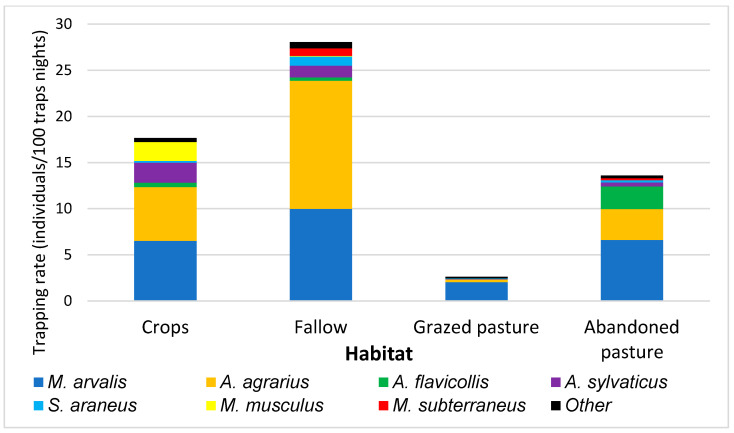
Trapping rates of the small mammal species in the four farmland types. Note: Trapping rates are expressed in terms of captured individuals per 100 trap nights. For a better representation, rare species (with less than six individuals captured in a habitat type) are considered together as Other.

Arable land had higher abundances compared to pastures, with the highest values recorded in fallow land, where *A. agrarius* was numerically dominant. Among the less common species, *Sorex araneus* (common shrew) and *Microtus subterraneus* (common pine vole) were best represented in this habitat (Figure 3). Crops were dominated by *A. agrarius* and *M. arvalis*, with similar trapping rates. *Mus musculus* (house mouse) and *Apodemus sylvaticus* (wood mouse) were also abundant. Grazed pastures had the poorest communities, with the lowest abundance and diversity (Table 2, Figure 3), represented mainly by *M. arvalis*. Grazing cessation, by favouring a rich herbaceous layer, led to a great increase in the occurrence of *M. arvalis* (still dominant) and *A. agrarius.* Meantime, by favouring the development of shrubs, it allowed for the presence of *A. flavicollis*, which was absent in grazed pastures (Table 2, Figure 3). The effect of grazing abandonment was different in the two specialists, i.e., the grassland specialist *M. arvalis* and the forest specialist *A. flavicollis* (Fisher’s exact test, *p* < 0.001). Both species benefitted from grazing cessation, but the increase in abundance was significantly stronger for the forest specialist.

### 3.2. Influence of Land Abandonment

Abundance and diversity were significantly higher in the abandoned farmland compared to the land in use. For diversity parameters, the effect of interaction between land abandonment and use type was not significant (Table 3). For abundance, in the model without interaction, the effect of abandonment was highly significant (χ^2^ = 20.46, d.f. = 1, *p* < 0.001), but its main effect became not significant when including the significant effect of interaction (Table 3). This means that the overall effect of abandonment on abundance is mainly caused by the increase in density when pasture grazing ceases. Analyses on the rodents alone produced very similar results (Table A1).

**Figure 4:**
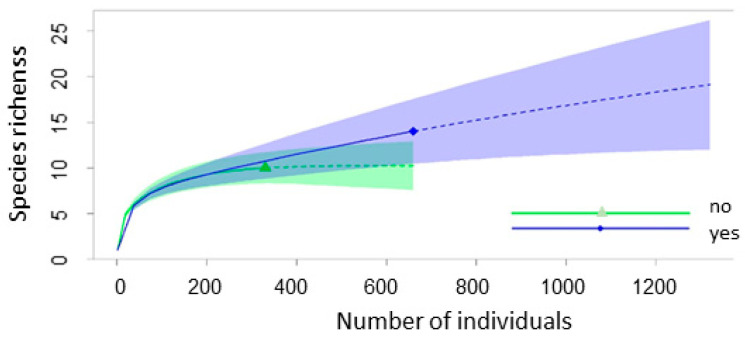
Rarefaction curves based on the number of captured animals of small mammal species depending on the land abandonment. Note: The blue line is for abandoned arable plots and pastures (yes), and the green line is for crops and grazed pastures (no). Shadowed areas represent the 95% confidence intervals of the estimated number of species.

Rarefaction curves show that abandoned plots have higher overall species richness (Figure 4). The sampling species saturation was reached in crops and grazed pastures but not in abandoned land, despite the two-times larger number of captured individuals. This was caused by the high number of rare species (singletons and doubletons) in the abandoned land, indicating that a larger sample size is needed to evaluate true species richness. Although the number of rare species was higher in grazed pastures compared to abandoned pastures (four compared to three), most of them overlapped with the species captured in crops, while in abandoned pastures rare species were complementary to those from fallow fields (Table 2).

**Figure 5:**
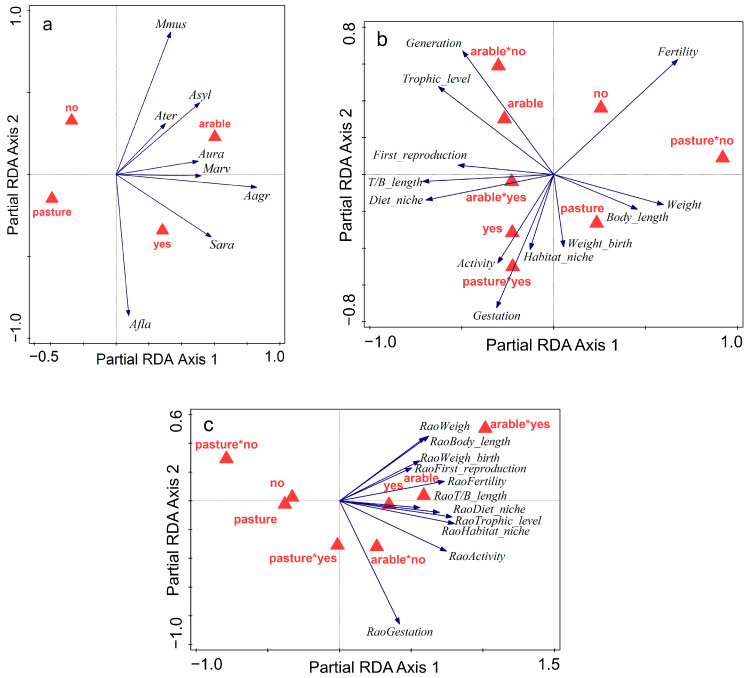
Biplots of partial RDA, summarizing the effect of abandonment (yes and no) in the two main types of farmland uses (pasture and crop) on (**a**) species composition, (**b**) functional community composition, expressed in terms of community weighted means, and (**c**) functional diversity. Note: Red triangles represent the centroids for the farmland types, arrows represent the direction in which the values of the response variables (species abundance, weighted means of traits, and Rao indices, respectively) increase, and * denotes interaction between the abandonment and land use type. Species are coded by the initial of the genus name and the first three letters of the species name. Codes of functional traits are given in the text, in the Materials and Methods section. Functional diversity is expressed as Rao index calculated for each functional trait. Station and month are included as covariates.

**Figure A2:**
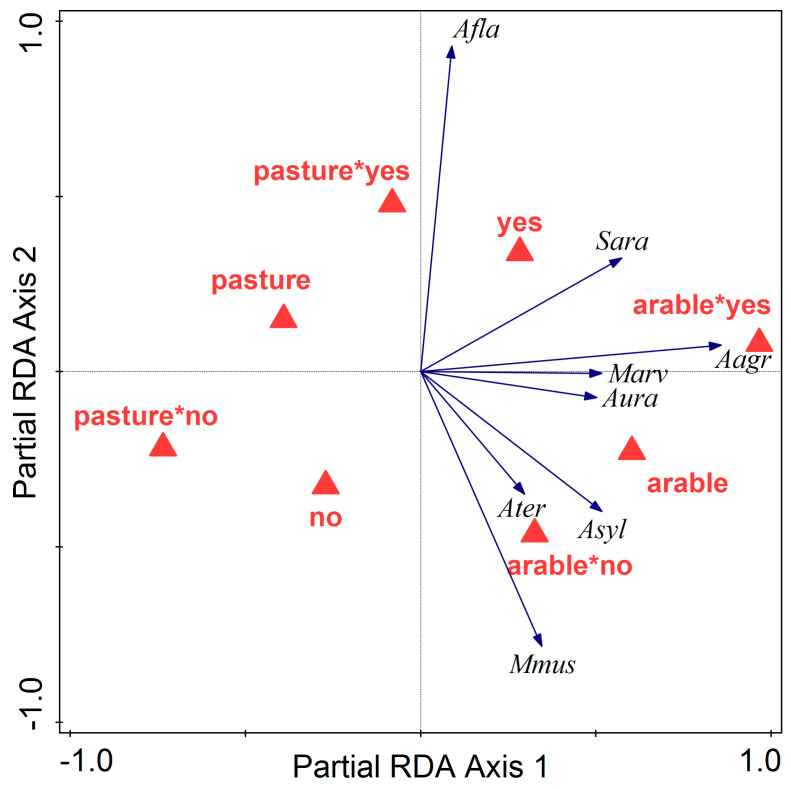
Biplot of the partial redundancy analysis (RDA) with station and month included as covariates, summarizing the effect of abandonment (yes and no) in the two main types of farmland uses (pasture and crop) on species composition. Note: * denotes interaction between the abandonment and land use type. Species are coded by the initial of the genus name and the first three letters of the species name.

**Figure A3:**
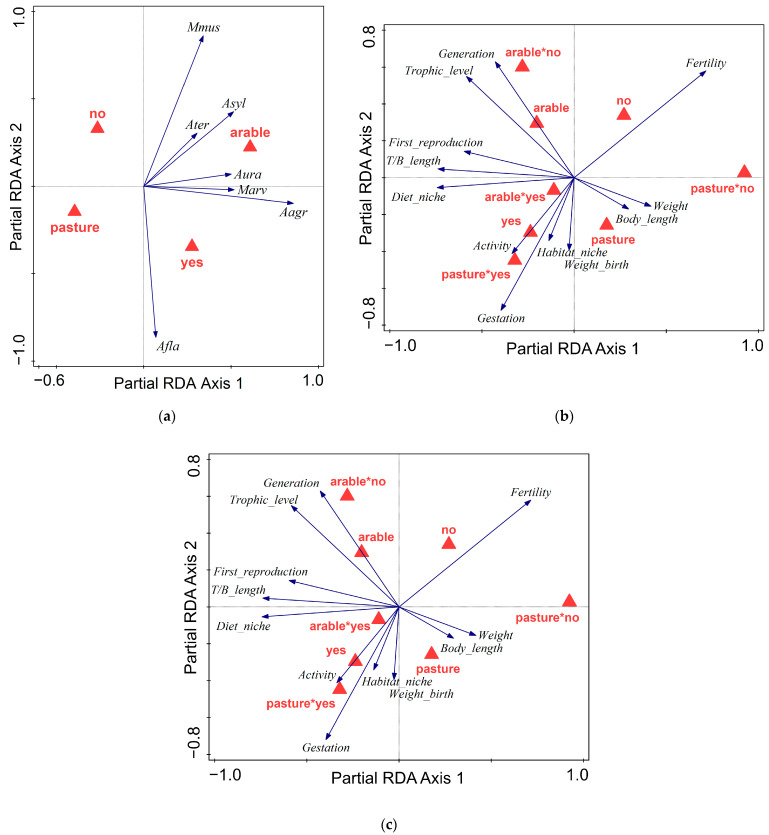
Biplots of partial RDA (station and month included as covariates), summarizing the effect of abandonment (yes and no) in the two main types of farmland uses (pasture and arable) on rodent (**a**) species composition, (**b**) functional community composition, expressed in terms of the community weighted mean (CWM), and (**c**) functional diversity, expressed as Rao index calculated for each functional trait. Note: Red triangles represent the centroids for the farmland types, arrows represent the direction in which the values of the response variables (species abundance, weighted means of traits, and Rao indices, respectively) increase, and * denotes interaction between the abandonment and land use type. Species are coded by the initial of the genus name and the first three letters of the species name. Codes of functional traits are given in the text, in the Materials and Methods section. Functional diversity is expressed as Rao index calculated for each functional trait. Station and month are included as covariates.

Taxonomic community composition had a significant (pseudo-F = 12.8, d.f. = 2, *p* = 0.001) and strong (24.1% explained partial variation—22.3% adjusted) response to the combined effect of land abandonment and use type. The first ordination axis was given mainly by the land use type, with most species being more abundant in arable land. The second ordination axis was given mainly by land abandonment, with *M. musculus* showing a significant preference for used farmland (crops), whereas *A. flavicollis* and *S. araneus* showed a strong preference for abandoned farmland (Figure 5a). *Microtus arvalis* and *A. agrarius* also showed a significant positive response to land abandonment (t-value biplot not shown). Both constrained axes were significant (pseudo-F = 11.1, d.f. = 2, *p* = 0.001, partial explained variation = 21.03% for the first axis, pseudo-F = 3.4, d.f. = 2, *p* = 0.034, partial explained variation = 3.06% for the second axis). Adding the interaction between land abandonment and use type to the model did not alter the responses of species composition (Figure A2) or the model quality (significance or explained variation). Including only the rodents in the analysis, the model was similar, with land abandonment and use type explaining 24.4% (22.6% adjusted) of the partial variation (pseudo-F = 13.4, d.f. = 2, *p* = 0.001) (Figure A3a).

By contrast, the interaction had a significant effect when analysing the response of the community functions. The partial RDA with the CWM in relation to land abandonment, use type, and their interaction (pseudo-F = 4, d.f. = 3, *p* = 0.005, 11.7% explained partial variation) was a better model than without the interaction (pseudo-F = 3.9, d.f. = 2, *p* = 0.007, 7.8% explained partial variation). Communities in fallow fields and abandoned pastures were characterised by a lower mean number of offspring produced and a reduced gestation length. Farmland abandonment favoured habitat generalists with more diurnal activity patterns, this effect being stronger in pastures than in arable land. Mean body size (weight, body length, and weight at birth) was larger for the communities in pastures compared to arable land, while the generation length and trophic level were lower, with a smaller difference for those in fallow fields (Figure 5b). The quality of the RDA model with the functional diversities in relation to land abandonment, use type, and their interaction (pseudo-F = 7.9, d.f. = 3, *p* = 0.001, 19.5% explained partial variation) was comparable with the model without the interaction (pseudo-F = 10.6, d.f. = 2, *p* = 0.001, 18.5% explained partial variation), but the response of functional diversities was altered. Diversity of body size (RaoWeight, RaoBody_length, RaoWeight) and reproduction (RaoFirst_reproduction, RaoFertility) was higher for the communities in arable land compared to pastures, and the difference was larger for those in abandoned farmland, especially fallow fields (Figure 5c). Including only rodents in the analyses, the models were similar and had higher explanatory power. Land abandonment, use type, and their interaction explained 15.6% (pseudo-F = 3.9, d.f. = 3, *p* = 0.003) of the partial variation in the functional structure (Figure A3b) and 25.1% in functional diversities (pseudo-F = 9.2, d.f. = 3, *p* = 0.001) (Figure A3c).

## 4. Discussion

We surveyed small mammal communities in agricultural habitats in used and abandoned land in two areas of southern Transylvania, Romania. In Hârtibaciu Plateau we recorded data on habitat characteristics (mainly vegetation attributes) for each surveyed plot, and we evaluated their effects on small mammal species composition and abundance [17]. Here, we expanded our research assessing the effect of arable land fallowing and grazing abandonment on the taxonomic and functional parameters of small mammal communities. In all, we captured 16 species. To the best of our knowledge, this is the highest number of species reported in a local study in Europe based on live trapping. The same species richness was found in Russia, in a mix of meadows, tillage, and fallow land of various ages in the southeast Tver Oblast region, over a 12-year period [40]. By comparison, a long-term study conducted over 30 years in northeastern Poland, recorded the presence of 12 small mammal species [20]. Although the results of the various studies are not directly comparable because of the differences in sampling design, other studies in southern and central Transylvania have confirmed that the area is home to diverse small mammal communities. Our results showcase mostly similar overall abundances but a much higher species richness compared to western and even central Europe, comparable with other regions in eastern Europe (Table A2). The rarefaction curves show that the species richness saturation has not been reached; abandoned crops may harbour even more species. Given that the type of trap and bait we used is more adequate for rodents than shrews, resulting in lower trappability of the latter, the abundance and occurrence of shrews in the study areas are probably underestimated. Therefore, we suppose that *S. minutus* and *C. suaveolens*, captured exclusively in cultivated crops or pastures in use, are very probably also present in the abandoned land. *Mus spicilegus* (steppe mouse)*, Microtus agrestis* (short-tailed field vole), and *Dryomys nitedula* (forest dormouse) were not trapped during the study, but are cited in the area [41,42,43] in similar habitats. Therefore, it is possible that they also inhabit the study area and could further increase the species richness of the abandoned land by increasing the trapping effort.

Our results supported the hypothesis that farmland abandonment is positively and significantly related to small mammal abundance and diversity, both taxonomic and functional (Table 3), similar to the other studies [10,21,44]. In one study, Janova and Heroldova [10] emphasised the high value of the set-aside land. By contrast, other studies have demonstrated that the effects of abandonment and agricultural practices are strongly influenced by landscape context, with the presence of habitat margins and surrounding farmland types playing a crucial role in how small mammals respond to abandonment [21,44]. Our results suggest that regions with a less intensive agricultural history and greater land fragmentation (such as Transylvania) may support higher small mammal diversity even in cultivated habitats, with abandonment likely further amplifying this diversity (Table 2, Figure 3). This result is somewhat similar to results from western Europe, showing that, in complex landscapes, small mammal abundance is higher compared to conventional croplands, suggesting that semi-natural habitats in these landscapes may offset the negative effects of intensive agriculture [44].

Compared to western and central Europe, farmland in Transylvania retains a higher species richness of small mammals due, on the one hand, to the high fragmentation of agricultural plots and, implicitly, the diversity of crops; on the other hand, the high species richness is also explained by traditional farming practices, partly still preserved in this area. The researched cropland harboured nine species (Table 2), more than the less intensively used plots in western and central Europe. For example, ref. [10] reported only seven species in the set-asides. In our research area, small plots can benefit from immigration from neighbouring plots even if they are under intermittent stress from different agricultural practices [6,23].

In western and central Europe—France [21], Spain [45], Italy [8], Czechia [10], Slovakia [6]—small mammal communities in the arable land are dominated by *A. sylvaticus* or *M. arvalis*. In eastern Europe, in Ukraine, the dominant species was *Apodemus uralensis* (pygmy field mouse) and, in some seasons, *A. sylvaticus* [46], which almost mirrored the findings in the Republic of Moldova, where the dominant species was *A. sylvaticus*, followed by *A. uralensis* [47]. In our study, although *M. arvalis* was the dominant species overall, its abundance was much higher in abandoned farmland, showing a preference for fallow fields rather than annual crops. The high abundance of *M. arvalis* in cropland in western and central Europe may show an adaptation of this species to annual and perennial alfalfa crops in the context of a sharp plummet in the availability of more natural habitats that the species typically inhabits.

In eastern Europe, we did not find similar work on the effect of grassland abandonment on small mammal communities. Our study shows the particular importance of grazing abandonment, indicated by the significant effect of the interaction between abandonment and farmland type on small mammal abundance. The more intense effect of abandonment in pastures compared to crops is due to the very low abundance and richness in grazed pastures, with the reduced anthropic pressure having a stronger effect. Pastures undergo increased stress throughout the grazing season, and this is evidenced both in terms of species richness (maximum two species per transect compared to the overall average of seven species per transect) and abundance (on average 2.6 individuals/100 trap-nights for pastures, compared to 14.8 individuals/100 trap-nights overall) (Table 2). Intensive grazing by livestock negatively affects wildlife due to habitat degradation. It alters habitat structure and food availability or quality by removing plant biomass, decreasing the height or cover of the herbaceous layer, and altering or changing the relative abundance of the different plant species [48,49]. Structurally simpler grazed areas also impact foraging and burrowing opportunities, and increase competition and predation risk [50]. Abandoned pastures have high ground cover, diverse plant communities, and the presence of woody vegetation. These provide food and protection and allow access to woodland species, such as *C. glareolus* and *M. avellanarius*, captured exclusively in these habitats. The strong positive response of small mammals to grazing cessation or reduction we found in the research areas is consistent with the findings from the studies elsewhere [48,50,51].

The studies involving various taxonomic groups show that the effect of grazing is dependent on the intensity of grazing and the successional stage. Because of the low and homogeneous sward structure, overgrazed grassland does not allow for the completion of the life cycles for many insects [52], resulting in resource-poor habitats for many passerine birds [52] and insectivorous mammals [53]. By contrast, light grazing increases vegetation heterogeneity, having a positive effect on small mammals, including increased shrew abundance [53] and rodent fertility [54]. However, the complete abandonment of grazing for long periods of time induces meadow-to-forest successions characterised by a decrease in small mammal diversity [55]. Unfortunately, we did not evaluate the time of the abandonment, which may be highly influential, as the responses of the small mammal communities to environment during the succession following pasture abandonment are not linear [56,57]. A likely scenario is that, if grazing is not resumed on these pastures, at a certain point, when the area becomes overgrown with woody vegetation, there will be only a few species adapted to such habitat, notably *A. flavicollis*—the only species captured in the neighbouring forests [17]. In addition, as mentioned in the description of the study areas, abandoned pastures were also occasionally grazed. With these limitations, our results illustrate the difference between intensely grazed pastures and those that had not been recently (during the previous 10 years) grazed or were only occasionally grazed. To further explore the responses of small mammals to pasture abandonment, and to disentangle their mechanisms, future studies should design the total exclusion of grazers and record the time of grazing cessation while also quantifying the grazing intensity in the used pastures.

Annual crops benefit from resting periods free from (or with low) anthropogenic stress between the agricultural work and harvest, providing small mammals with both habitat and food resources, as evidenced by species richness (maximum six species per transect) and the number of individuals (average 13.5 individuals/transect) (Table 2). Besides the traditional way of farming in the area, the high diversity and abundance are also explained by the fact that this study was conducted between June and October, when crops provide sufficient food and shelter resources. During this period, stress-inducing agricultural work, such as ploughing or the deployment of pesticides, no longer takes place in most crops, allowing for these habitats to support abundant and diverse small mammal assemblages. Our results indicate their high resilience in small plots, as they are able to rapidly return to their original state after the stress caused by the agricultural work. This resilience is facilitated by their immigration from undisturbed plots, which act as refuges during farming activities.

By abandonment, diversity and abundance in the arable land increased significantly, with fallow fields having the highest capture rates and species richness, consistent with those of the other studies in Europe [10] and beyond [58]. This is due to high ground cover, which ensures protection from predators, with lush and diverse herbaceous vegetation yielding a great variety of both plant and invertebrate food resources, which provide niches for many small mammal species [50]. Larger differences between crops and fallow fields might have been detectable if the sampling had included periods of peak agricultural activity (e.g., soil tillage, sowing, harvesting). It is conceivable that, during the sampled period, small mammals were abundant in the surveyed crops, whereas during intensive farming phases, they may have sought refuge in field margins or fallow land.

Taxonomic species composition was affected mainly by the land use type, with most species preferring the arable land (Figure 5a). Here, the effect of temporary human disturbance through agricultural work is outweighed by the high environment heterogeneity. Responses to abandonment on the other hand, are more nuanced. *Mus musculus* was, in our study, the only species showing a significant preference for crops. It was captured mainly in maize fields without weeds close to villages, in relation to its synanthropic nature. Therefore, this species could be used as an indicator of human disturbance and intensification of agricultural land use. By contrast, the strongest positive overall response to abandonment was shown by *A. flavicollis*. Its abundance in arable land was not affected by fallowing, because of the lack of woody vegetation. The key factor for *A. flavicollis* in the farmland is the shrub encroachment in the abandoned pastures. These represent, the characteristic habitat for the yellow-necked mouse in the rural mosaic. Similarly to *A. flavicollis*, the dominant *M. arvalis* also had a stronger increase in abundance in the abandoned pastures. Both these species are specialists, but they are characteristic for different habitats. Their coexistence in large numbers in abandoned pastures is an indicator of the high biodiversity value of this habitat, with features favourable for both grassland and forest species. The prevalence of the grassland specialist confirms that the abandoned pastures in our survey were in an early successional stage. The positive response of *A. agrarius* was similar in the two land use types, suggesting it is more dependent on the presence of tall and abundant vegetation and less sensitive to food availability [50]. Among the insectivores, *S. araneus* showed a positive response, with the highest abundances in fallow land but also showing a significant increase in abandoned pastures, suggesting a drop in the presence and abundance of invertebrates in the grazed pastures [51]. From this, a greater shrew preference for less intensively used and qualitatively superior habitats [52,53] can be inferred, although these results may be biased by the difference in shrew trappability in our study.

The CWM trait value distributions vary with the habitat type. Partial RDA diagrams showed strong segregations of species groups along the first axis in relation with the farmland type, and along the second axis with abandonment (Figure 5b). The communities in arable land are characterised mainly by high trophic levels, long gestation, and small size. The high trophic level is given by the large share within the community of insectivorous and omnivorous species, supported by the abundant invertebrate populations in fallow fields. Because shrews are on the highest trophic level, this is associated with their small sizes. The high values of diet niche in fallow fields are probably related to the more complex habitat structure and composition. These ensure a wide spectrum of trophic (e.g., roots, leaves, seeds, insects) resources [25]. Habitat niches are also wider in the abandoned farmland, with the higher environmental heterogeneity providing a range of spatial niches [50]. In addition, *A. agrarius*, the species with the strongest response to abandonment, is a habitat generalist. The intensively used habitats, which are preferred by *M. musculus* and *A. sylvaticus*, are characterised by high fertility and shorter gestation. This corresponds to the r reproductive strategy associated with the disturbed habitats, unpredictable vital resources, and high risks adopted especially by the synanthropic *M. musculus*. The tail to body length ratios are large in scansorial mice (such as *A. flavicollis* or *M. minutus*), small ratios are an adaptation to fossoriality and are typical for voles, and intermediate values are characteristic for terrestrial mice (such as *A. agrarius*) or shrews. Because grazed pastures offer little nesting shelters or protection against predators, fossoriality is an important adaptation for survival in these habitats. Therefore, small mammals in grazed pastures are characterised by the smallest tail to body length ratios. Diurnal activity is lowest in used farmland, both arable and pasture, where the repeated human disturbance interferes with the animal activity patterns. Disturbed habitats, especially pastures, are also loosely associated with a larger body size (body length and weight), which may offer advantages for coping with unpredictable food availability and patchily distributed resources, as well as for predation deterrence [25].

Abandoned farmland is associated with highly heterogeneous functional traits, revealed by the larger values of the Rao functional diversity indices calculated separately for each trait. The high taxonomic diversity found in the abandoned cropland entails a high diversity of most functional traits (Figure 5c), with species living in fallow fields having more different traits. This means greater trait variability within these communities that result in a wider range of ecological strategies to cope with environmental challenges. Land fallowing has been shown to restore soil fertility through an increase in organic matter, nutrient deposits, microbial carbon, and overall microbial diversity and activity. It has also been proven to reduce pest populations (weeds and insects) by disrupting their life cycles, limiting the reliance on pesticides, and helping to mitigate climate change by reducing the emissions of greenhouse gases and increasing the carbon sequestration in the soil [9]. The high functional diversity we found in fallow fields indicates that the discontinuation of land cultivation may have additional benefits, due to the more functionally diverse small mammal communities, which may provide other ecosystem services. For example, a high diversity of tail to body length ratio (used as a proxy for arboreality) means that terrestrial species live alongside subterranean species, which contribute to bioturbation [59,60], while a high trophic level diversity indicates the coexistence of species with various diets, contributing to seed dispersal, as well as the predation [61,62,63] and consumption of pest invertebrates [64].

Previous studies have found that small mammals (alongside insects and birds) benefit from set-asides in intensive agricultural landscapes, while in complex landscapes, their beneficial effects are negligible [10,13]. Our results show that small mammal communities may benefit from short-term farmland abandonment even in complex mosaic landscapes, in the context of a rich species pool that renders high overall biodiversity. As stated before, due to our trapping design, the abundance and occurrence of shrews are probably underestimated. Given that the models on functional structure and diversity that included only rodents had a higher explanatory power compared to those for the whole small mammal community, we may assume that further studies better suited for shrews would reveal an even stronger effect of farmland abandonment on small mammals.

Based on our results and previous works, we can make some biodiversity conservation recommendations specific to agricultural habitats. Given the peculiarity of our study area, our recommendations are tailored for this region and may not be suited for other areas. We recommend the maintenance of uncultivated plots, which helps boost biodiversity [10]. Our results also reinforce the recommendation of Schmidt et al. [53] regarding agricultural schemes, stating that low-intensity grazing and woody vegetation are desirable where grazing is imposed. Therefore, where the high number of livestock do not allow for low-intensity grazing, we recommend the introduction of a scheme in which intensively grazed habitats undergo resting periods that would favour the recovery of the vegetation. The restored vegetation would support abundant small mammal communities, including local populations of less abundant species (e.g., *S. minutus*, *C. leucodon*, *A. uralensis*), in addition to the predatory birds and mammals relying on these prey species [3,65]. Although we did not directly test the beneficial effects of the mosaic character of the landscape, our results suggest this. Therefore, in addition, we propose the maintenance of small-sized crops by encouraging the villagers to keep and work their land in a traditional manner, thus contributing to the high background diversity. All of these measures may be implemented trough a subsidy scheme and the development of ecotourism, the study area being already one of the most important ecotourist regions in Romania.

## 5. Conclusions

Our study compares the abundance, the species richness, and the taxonomic and functional diversity of small mammal communities in used and abandoned farmland. In accordance with our first hypothesis, diversity and abundance were significantly higher in the latter. Our second hypothesis, stating the different changes in community composition between arable land and pastures was partly confirmed, for the functional composition but not for species composition. In accordance with our third hypothesis, *A. flavicollis* increased in abundance following grazing abandonment, partially replacing *M. arvalis* in the community composition. The surveyed area—southeastern Transylvania—harbours, even in intensively-used habitats (crops and grazed pastures), species-rich small mammal communities, much more diverse compared to the farmland in western and central Europe, and similar to those from other parts of eastern Europe. This is probably due to the high fragmentation of agricultural plots, with profound implications for their ability to maintain biodiversity. Species and trait distributions varied along with the different farming management types, highlighting the need to preserve mixed mosaics that can support high taxonomic and functional small mammal diversity. These are prerequisites for viable and valuable ecosystem services, such as bioturbation, seed dispersal, and pest control. Small mammals are also an important prey for farmland predators. Our results highlight that the local temporary abandonment of arable land and pastures is a farming management measure with a positive effect on small mammal abundance, richness, and functional diversity, which are indicators of agricultural landscape integrity and the degree of anthropisation.

## Figures and Tables

**Table 1:** Trapping effort (effective trap nights) and number of transects (in parentheses) in the four farmland types in the study seasons.

Season	Crops	Fallow	Grazed Pasture	Abandoned Pasture	Total
Autumn 2010	258 (5)	412 (6)	348 (6)	487 (7)	1505 (24)
Summer 2011	737 (9)	485 (5)	511 (7)	643 (11)	2826 (32)
Autumn 2011	618 (7)	436 (5)	864 (14)	908 (12)	2376 (38)
Total	1613 (21)	1333 (16)	1723 (27)	2038 (30)	6707 (94)

**Table 2:** Results of trapping in relation to abandonment (no—land in use, yes—abandoned land).

Species	Arable Land		Pasture		Total
	No	Yes	No	Yes	
*Microtus arvalis*	105	133	35	135	408
*Apodemus agrarius*	94	185	5	68	352
*Apodemus flavicollis*	8	5	0	59	72
*Apodemus sylvaticus*	35	17	1	9	62
*Sorex araneus*	3	13	1	5	22
*Arvicola terrestris*	1	2	1	0	4
*Mus musculus*	33	1	0	0	34
*Apodemus uralensis*	5	5	0	3	13
*Microtus subterraneus*	0	11	0	4	15
*Micromys minutus*	0	1	0	0	1
*Sorex minutus*	0	0	2	0	2
*Crocidura suaveolens*	1	0	0	0	1
*Crocidura leucodon*	0	0	0	1	1
*Clethrionomys glareolus*	0	0	0	1	1
*Muscardinus avellanarius*	0	0	0	1	1
*Rattus norvegicus*	0	1	0	0	1
Number of species	9	11	6	10	16
Number of species per transect (min-max)	0–6	2–7	0–2	0–4	0–7
Chao (min–max)	0–6	2–8	0–3	0–8	0–8
Shannon (mean value per transect)	0.709	0.881	0.111	0.466	
Rao (mean per transect)	0.207	0.291	0.042	0.156	
Number of individuals	285	375	45	286	991
Total abundance (individuals/100 trap-nights)	17.7	28.1	2.6	14	14.8

**Table 3:** The best univariate models for the parameters of the small mammal community.

Coefficients	Estimate	Std. Error	Test Statistic	*p*
**abundance**			z	
intercept	1.791	0.37		
pasture	−2.23	0.322	−6.194	<0.001
abandonment	0.319	0.299	1.065	0.286
pasture * abandonment	1.429	0.429	10.29	<0.001
marginal (and conditional) R^2^	0.462 (0.733)			
**species richness**			t	
intercept	2.611	0.248		
pasture	−1.827	0.281	−6.5	<0.001
abandonment	1.211	0.274	4.407	<0.001
R^2^ (adjusted)	0.371			
**Chao**			t	
intercept	2.929	0.307		
pasture	−2.097	0.348	−6.024	<0.001
abandonment	1.412	0.34	4.152	<0.001
R^2^ (adjusted)	0.337			
**H**			t	
intercept	0.66	0.072		
pasture	−0.512	0.082	−6.225	<0.001
abandonment	0.284	0.08	3.351	<0.001
R^2^ (adjusted)	0.329			
**Rao**			**t**	
intercept	0.256	0.027		
pasture	−0.202	0.031	−6.441	<0.001
abandonment	0.135	0.03	4.421	<0.001
R^2^ (adjusted)	0.368			

Note: Community parameters (response variables) include abundance (total abundance of small mammal communities), species richness (number of trapped species), Chao (estimated number of species), H (Shannon index), Rao (functional diversity) abandonment and land use type (pasture) are predictors, with crops as the reference level. Note: * denotes interaction between abandonment and land use type. The abundance model, using the negative binomial distribution with a log link function, includes station and month of the survey as random factors. All the other models are linear. Marginal pseudo-R^2^ is the variation accounted for by the fixed effect in the mixed model, and conditional pseudo-R^2^ is the variation accounted for by fixed and random effects.

## Data Availability

All data used for the analyses presented in the manuscript are available as Supplementary Material.

## Supplementary Materials

The following supporting information can be downloaded at: https://www.mdpi.com/article/10.3390/ani15131857/s1, Table S1: Number of small mammal individuals captured in relation with the land use type and abandonment.

## Abbreviations

The following abbreviations are used in this manuscript:

CAP	Common agricultural policy
GLMM	Generalised linear mixed model
RDA	Redundancy analysis
CWM	Community weighted mean

## Appendix A

**Table A1:** The best univariate models for the parameters of the rodent community.

Coefficients	Estimate	Std. Error	Test Statistic	*p*
**abundance**			z	
intercept	1.785	0.373		
pasture	−2.299	0.327	−7.029	<0.001
abandonment	0.281	0.3	0.935	0.349
pasture * abandonment	1.515	0.432	3.502	<0.001
marginal (and conditional) R^2^	0.47 (0.738)			
**species richness**			t	
intercept	2.442	0.225		
pasture	−1.675	0.255	−6.572	<0.001
abandonment	0.976	0.249	3.918	<0.001
R^2^ (adjusted)	0.36			
**Chao**			t	
intercept	2.62	0.267		
pasture	−1.862	0.303	−6.149	<0.001
abandonment	1.159	0.296	3.917	<0.001
R^2^ (adjusted)	0.336			
**H**			t	
intercept	0.594	0.069		
pasture	−0.479	0.078	−6.12	<0.001
abandonment	0.271	0.076	3.546	<0.001
R^2^ (adjusted)	0.324			
**Rao**			t	
intercept	0.187	0.022		
pasture	−0.152	0.025	−6.141	<0.001
abandonment	0.109	0.024	4.504	<0.001
R^2^ (adjusted)	0.356			

Note: abundance (total abundance of rodent communities), species richness (number of trapped species), Chao (estimated number of species), H (Shannon index), Rao (functional diversity) considering abandonment and land use type (pasture) as predictors, with the crops as the reference level. Note: * denotes interaction between abandonment and land use type. The abundance model, using the negative binomial distribution with a log link function, includes station and month of the survey as random factors. All the other models are linear. Marginal pseudo-R^2^ is the variation accounted for by the fixed effect in the mixed model, and conditional pseudo-R^2^ is the variation accounted for by fixed and random effects.

**Table A2:** Results of studies on small mammals (number of captured individuals and species) conducted in various parts of Europe in agricultural or mixed landscapes, considering the type of surveyed habitats, the trapping effort (trap-nights), and the method.

Country	Region	Individuals (Species)	Surveyed Habitats	Trapping	Reference
Romania	S Transylvania, Hârtibaciu Plateau and Făgăraș Piedmont	991 (16)	corn, wheat and potato crops, fallow fields, pastures, abandoned pastures	6707 (live traps)	present study
Romania	S Transylvania, Hârtibaciu Plateau	136 (13)	mix of agricultural (hayfields, corn and potato crops) and non-agricultural (forests, clearcut, reedbed) habitats	418 (live traps)	[41]
Romania	Central Transylvanian Plain	200 (8)	sessile oak-hornbeam woodland, meadow, pasture, pasture with shrubs, oak woodland, forest edge	1200 (live traps)	[42]
Romania	S Transylvania, Râul Negru River Basin	192 (9)	cemetery, churchyard, river bank, crops	1654 (live traps)	[18]
Slovakia	Nitra, SW Slovakia	125 (5)	wheat, peas, and grass mixture crops in the conditions of ecological farming and barley, rapeseed, and maize crops	32,850 (pitfall traps)	[6]
Poland	north-eastern Poland, near Łuknajno Biosphere Reserve	not mentioned (12)	mosaic of agricultural and forest habitats comprising mixed forest, lakeshore alder forest, wheat, oat, rye, maize, and potato crops, which were abandoned during the second half of the study period	not mentioned (live-traps in a 30-year study)	[20]
Czech Republic	S Moravian Pannonian lowland	892 (9)	alfalfa, spring barley, maize, winter rape, sunflower, and winter wheat fields, steppe-type set-aside and old abandoned orchards in a mixed agricultural landscape	35,900 (snap-traps)	[10]
Czech Republic	S Moravia	5536 (14)	wheat, barley, maize, sugar beet, alfalfa, vegetables, tobacco, potatoes, sunflowers, windbreaks, small forests, fallow land, and vineyards.	51,480 (snap-traps)	[23]
Germany	S Lower Saxony	410 (11)	organic and conventional winter wheat field plots, covering a gradient of landscape complexity with arable land varying between 94% and 41%	3960 (live traps)	[44]
Germany	NE Germany, Brandenburg	455 (7)	Cereal fields, oilseed rape fields, grassland, forest, field margins, kettle holes	3840 (live traps)	[67]
Switzerland	C Switzerland, Wauwil	349 (6)	low-intensity meadows, wild-flower strips, and herbaceous strips, artificial grassland and autumn-sown wheat	5400 (live traps)	[22]
Italy	NE Italy, Friuli Venezia Giulia region	668 (4)	three study areas differentiated along a gradient of agricultural intensification	4630 (live traps)	[8]
France	Brittany, south of the Mont Saint-Michel Bay	1782 (8)	hedges, woods, and meadows	19,584 (live traps)	[3]
France	Brittany, Mont Saint-Michel Bay	>1523 (10)	agricultural edge habitats, such as dykes and hedgerows in an intensively cultivated landscape on a reclaimed area (polders)	9180 (live traps)	[21]
France	Brittany, south of the Mont-Saint-Michel Bay	1379 (8)	24 plots located in hedgerows	11,424 (live traps)	[2]
Ukraine	Slobozhanshchyna (eastern Ukraine)	380 (12)	ravine–gully systems, agroecosystems, field-protecting forest strips, riparian vegetation along artificial reservoirs and streams, dry meadows, and pastures	4900 (live trap)	[46]
Russia	central part of European Russia: in the vicinity of the Bakanovo village, the Staritsa Region of Tver Oblast’	1869 (10)	abandoned crops and pasture	not explicit (18-year study using live traps)	[57]
Russia	southeast Tver Oblast on the right-hand bank of the Volga River within the Smolensk–Moscow Province	4774 (16)	meadows, tillage and fallow land of various ages	not explicit (12-year study using live traps)	[40]
Republic of Moldova and Romania	central Moldova Bacău county	Bacău—11 species, Moldova—9 species	agroecosystems	not explicit	[68]
Republic of Moldova	central Moldova	9395 (13)	different types of biotopes, varying in their degree of heterogeneity and human impact	not explicit	[47]
